# An operational guide to resin 3D printing of geological macromodels

**DOI:** 10.1016/j.mex.2022.101863

**Published:** 2022-09-16

**Authors:** Mohamed Idris, Thomas Daniel Seers, Nayef Alyafei

**Affiliations:** Petroleum Engineering Program, Texas A&M University at Qatar, Education City, Doha, Qatar

**Keywords:** 3D printing, Modelling, Stereolithography (SLA)

## Abstract

Stereolithography (SLA) is a form of 3D printing that is based on the curing of resin under UV light. There are a wide variety of 3D resin printers on the market that all follow the same general procedure. First, a slicing program is used to slice the model in a sequence of thin layers. The model will be printed in this sequence of layers after it is exported in a format recognizable by a 3D printer. In addition to this main function, slicing programs offer additional features to manipulate the model, adjust print settings, and add model supports. Next, after the printer is set up, the sliced model is loaded onto the printer and fabricated. Once the print is complete, the model can be washed, cured and sanded/polished to the desired finish. In this work, we utilize SLA 3D printing to print geological macromodels, to be utilized in flooding experiments. Images captured from the flooding experiments were then incorporated in a set of visual learning exercises for undergraduate students to enhance the study of immiscible fluid flow in porous media. SLA printing was selected in this use case as it provides important advantages over other common 3D printing technologies (e.g. Fused Depositional Modelling: FDM), such as high print resolvability of sub-millimeter scale pore geometry and a high degree of transparency within the resultant printed models. Overall, this method was found to:

• Provide an engaging learning experience for undergraduate students, as the captured flooding experiment image time series allowed students to directly visualize often obtuse fluid flow processes in porous media.
• Be easily reproducible: after completing an initial print the method can be reproduced for many different pore networks, allowing for a wide array of comparative studies and learning exercises to be developed.

## Graphical abstract

**Figure:**
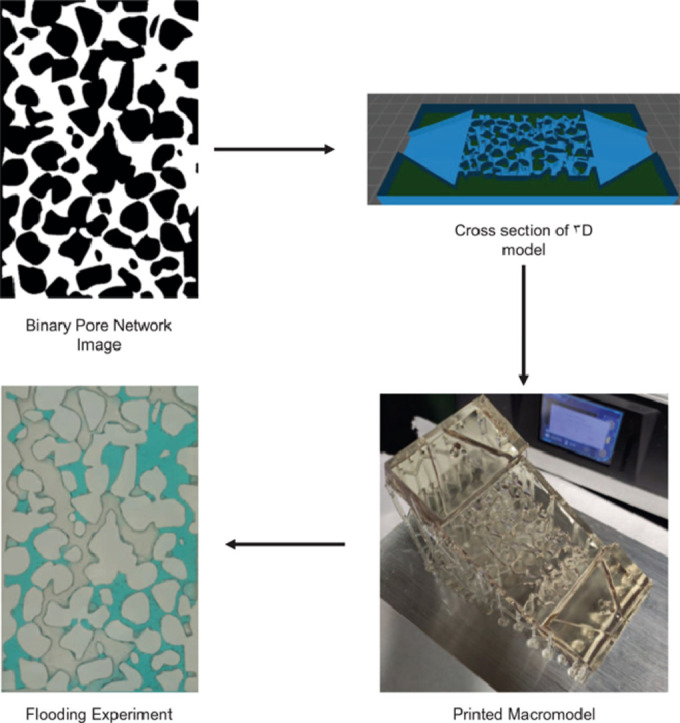

Specifications table

**Table:** 

Subject Area:	Engineering
More specific subject area:	*3D Printing*
Method name:	*3D printing of Geological Macromodels*
Name and reference of original method:	• General Resin 3D Printing Instructions • Reference: Shenzhen AnyCubic Technology Co. [9]. AnyCubic PhotonMono X: User Manual. • The original resin 3D printing method found in the referenced user manual was adapted to analyze and study geological macromodels.
Resource availability:	Slicing Software Download Links: Chitubox, Prusa_Slicer AnyCubic_PhotonMono_X AnyCubic_Wash_and_Cure_Machine

## Method details

**Fig. 1:**
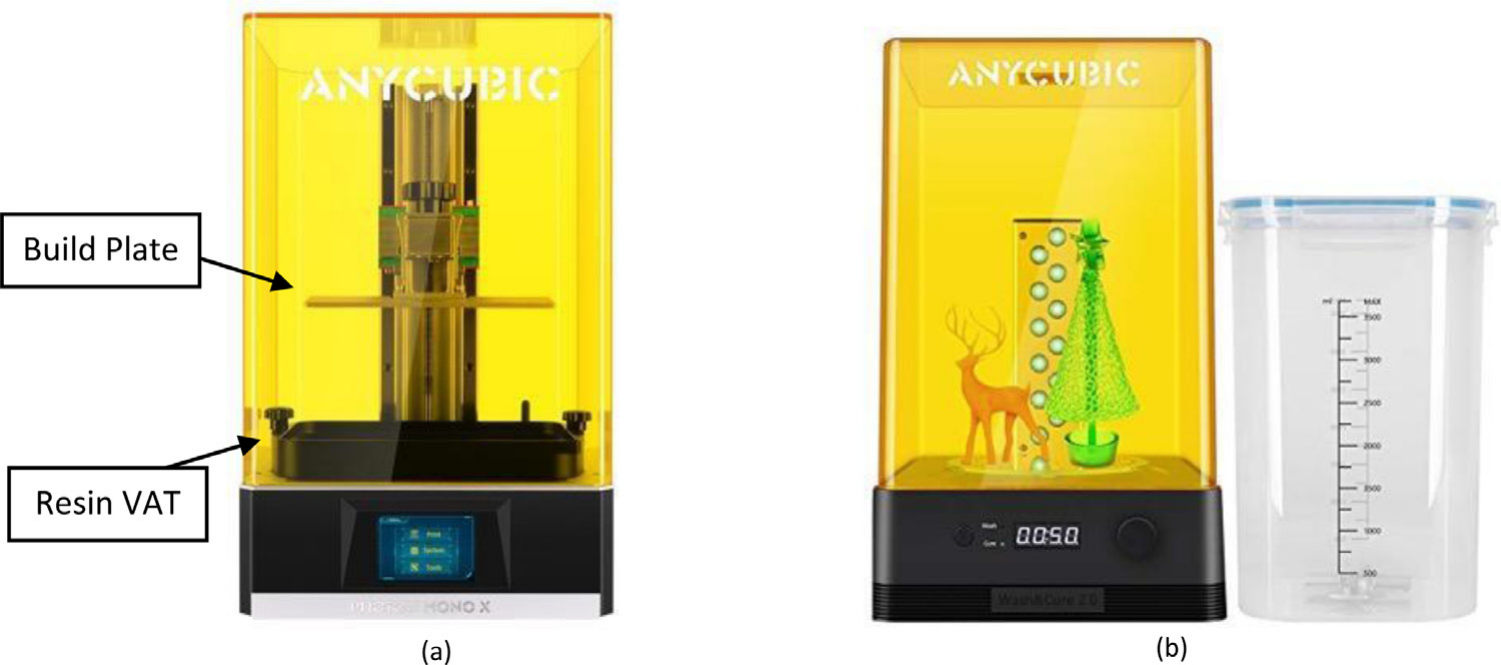
(a) The AnyCubic Photo Mono X used to 3D print the macromodels and (b) the AnyCubic Wash and Cure machine used to clean and cure the macromodels during post processing (Shenzhen AnyCubic Technology Co).

The SLA 3D printing workflow can be divided into two stages: (1) preparing the 3D model using a slicing software, and (2) printing and post processing the 3D model. Whilst there are a wide variety of commercial SLA 3D printers available on the market, the underlying principles to how these systems operate are equivalent. SLA printers use UV light to cure/harden resin layer-by-layer to create a 3D structure [4]. Therefore, the procedures outlined in this paper can be utilized irrespective of the printer used. The SLA 3D printer utilized throughout this paper is the AnyCubic PhotonMonoX (Fig. 1).

## Preparing the 3D print on a slicing software

**Fig. 2:**
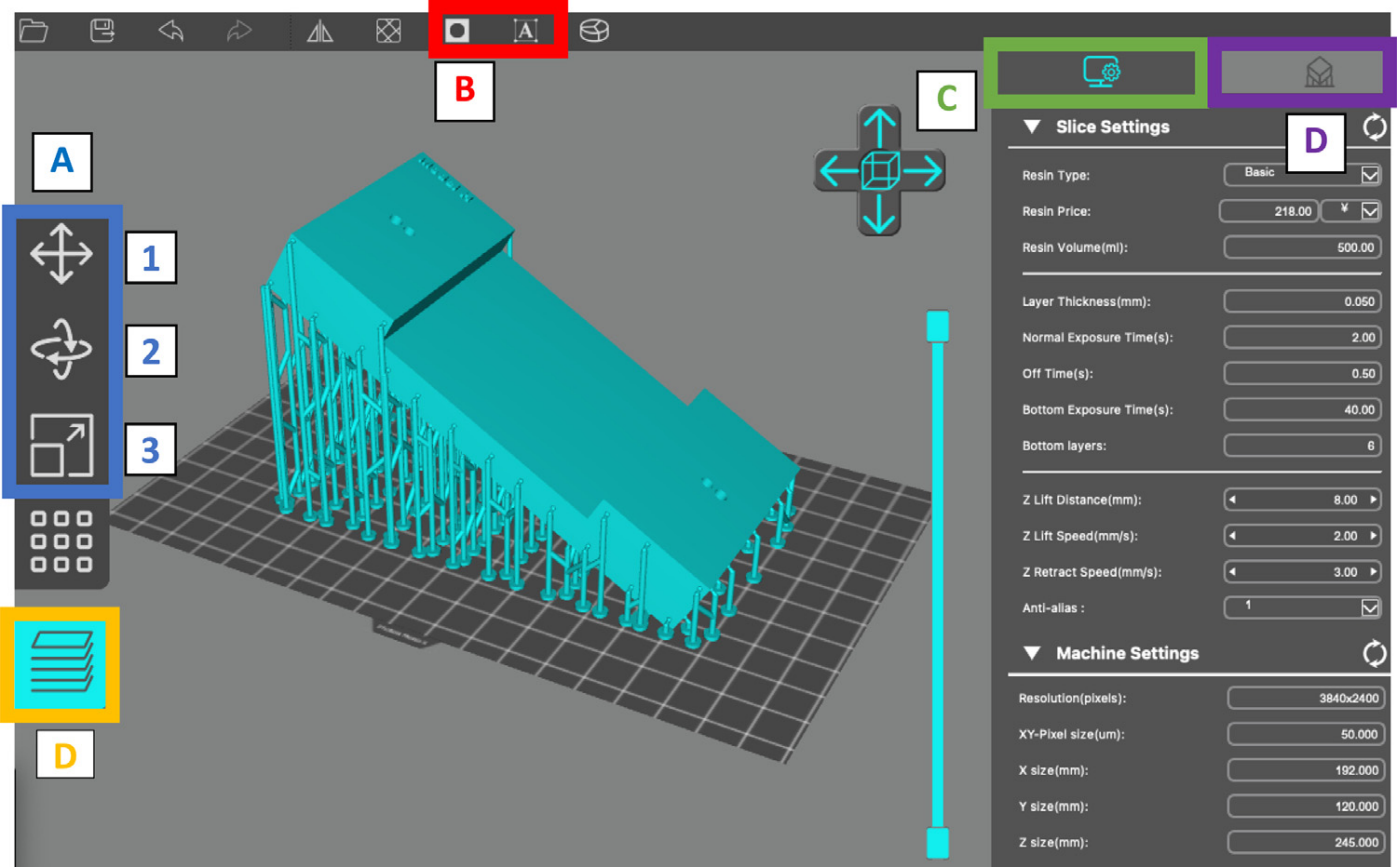
The Photon Workshop interface labelled with some of the software's main features.

Before a macromodel can be fabricated, a mesh-based representation of the porous media must be prepared using a slicing software and exported to a printable format. There is a wide variety of slicing software tools available. Typically, SLA printer manufacturers will include their own slicing software with their system. Additionally, there are several third-party slicer tools available, including Chitubox, Prusa Slicer and Photon Validator. The main function of slicing software is to discretize the model into a sequence of vertically stacked thin layers that can be parsed by the 3D printer. In addition to this main function, slicing software tools offer additional features to manipulate the scale and orientation of the model, adjust print settings, and add model supports required to stabilize the piece during fabrication. It is important to note that the slicing program has no intrinsic capacity for model design. Computer-aided design (CAD) software tools, such as AutoCAD are often used to design the models, which can be exported using the stereolithography (.stl) mesh exchange format. In addition, some authors have developed application-specific toolchains that facilitate the design of 3D printable proxy pore structures (e.g. [7]). It should be noted that mesh based representations of porous media generated by such methods are also amenable to computational fluid dynamic simulations (e.g. [5]). The 3D printer used in this paper is the AnyCubic PhotonMonoX and is provided with a proprietary slicing software (Photon Workshop) / (Fig. 2). In the following section we will provide an overview of the key functionality of Photon Workshop's graphical user interface (GUI) in relation to macromodel pre-processing for 3D print setup. The reader should note that equivalent functionality is available within most commercial slicer software tools.

**A, Model size and orientation:** The pushbuttons highlighted in the blue rectangle provide the ability to adjust the model's orientation, size, and location on the build plate. With reference to Fig. 2, Photon Workshop's spatial manipulations tools are detailed as follows:

(1) The top pushbutton opens a pop up that allows the user to translate the model along the coordinate frame's x,y and z axes by a specified number of millimeters on the build plate. This feature is useful when printing multiple models at once and ensuring each model has adequate space on the build plate. The pop up also allows the user to center the model. Additionally, the user can move the model by holding left click on the visualization and dragging the model. However, the pop-up tool allows for more precise positioning.
(2) The center pushbutton allows the model to be rotated along the x, y and z axes of the coordinate frame. This feature is important for ensuring 3D SLA prints print correctly. In fact, in most cases it is recommended that the model is printed at a 30-degree angle from the build plate. This oblique configuration ensures any uncured resin can be drained properly out of the model and minimizes the number of overhangs (portions of the model that hang off the build without structural support) or islands (portions of a model that are not in contact with other model components or the build plate) that can often lead to failed prints [4]. Generally, the most effective solution to mitigating the deleterious impact of overhangs and islands to print quality is to add supports to the build. However, such supports can reduce the final finish of the fabricated model: a key consideration for transparent builds. Adjusting the orientation of the print can minimize the number of overhangs/islands and consequently the number of supports required.
(3) The final highlighted button allows the user to scale the model in the x,y and z directions. Again, this an important feature that will easily allow users to resize the model quickly and easily. The popup window the button activates allows the user to scale in all three axes simultaneously by the same factor or scale along each axis individually.

**B, Toolbar features:** Along the toolbar are two highlighted features. From left to right these are:

(1) **Hole punching:** The second feature allows a hole to be punched through the model. This feature is mainly used to create drain holes for hollow models. Generally, two drain holes at the bottom of the model are required. These allow for uncured resin to flow out the model during the print and also provide an ability for isopropyl alcohol to flow into the model during post-processing.
(2) **Text paste:** The final feature to note is the ability to overlay text onto any surface of the model. This feature is useful when models need to be easily distinguished from one another. For instance, the feature can be used to label printed models when trialing different print settings or similar pore network geometries.

**C, Slice settings:** To the right of the graphical user interface are two sections that can be toggled. The first provides the user with slice/print settings that can be adjusted. Generally, the default values for these settings are fairly optimized and result in successful prints. However, these settings can be adjusted in order to obtain more optimal results. These settings include:

(1) **Layer thickness:** This setting adjusts the thickness of each of the slice layers. A smaller layer thickness will increase print times but capture a higher level of detail, whilst a larger layer thickness will reduce print times and reduce the level of detail. 0.05 mm is considered as the standard value for layer thickness whilst 0.01 mm is defined as ultra-fine [6].
(2) **Exposure times:** The time (seconds) that the printer will expose each layer to UV light during printing. Exposure times can affect the quality of the print as different resins/printers require different curing times. Optimal exposure times for particular resins/printers can usually be found in the manuals that accompany a printer/resin. Generally, a 6 s exposure time is sufficient for most resins. A bottom exposure time can be set for the first few layers at the base of the model. The number of layers considered as ‘bottom layers’ is also user defined. It is recommended that bottom exposure time be roughly 8–12 times longer than regular exposure time. A longer exposure time is required for the lowermost layers to ensure the base of the print adheres to the build plate (Resin 3D Printer: Settings Guide).
(3) **Z lift speed and distance:** The Z lift speed refers to the speed at which the build plate is lifted from the resin VAT/tank. Increasing the value of this setting will reduce print times but also adds strain to the model's connection to the build plate. If the Z lift speed value is too high, the build may become detached from the build plate. Generally, a Z lift speed of 1–2 mm/s is recommended. Z lift distance is the distance the build plate raises up from the UV screen film after each layer is cured. Generally, this setting should be kept as default and does not require adjustment (Resin 3D Printer: Settings Guide).

**C, Print supports:** Adding an adequate number of supports at appropriate locations is essential towards the creation of successful prints. This is particularly true when the model has long overhangs or islands. Supports provide structural support to such regions of the model and enhance the build's contact area with the build plate. Supports can vary in size and shape depending on the model's requirements and the nature of the overhangs. Thicker supports can be used for excessive overhangs while smaller supports can be used in minor overhangs. The disadvantage of using thicker supports is they require greater effort to remove and take up a larger footprint on the model which can lead to spurious features upon the model surface if not properly removed. It is thus recommended to minimize or omit supports on the macromodel's cover slip (e,g. Fig. 2). Supports can be added or removed manually or automatically, whereby the slicer software places supports in model regions where the need for additional support is detected. It is critical that supports are not present within the macromodel's pore structure, as this would have a deleterious impact upon the targeted flow experiments. The accuracy to which automatic supports are placed varies based upon the slicing software, however it is generally recommended to add automatic support and then add or remove manual supports in any remaining problem areas for best results [3].

## Printing and processing procedure

Once the sliced model has been exported to a removable drive, it can be imported into the SLA printer, typically via a USB port. Generally, the following generic steps must be completed to execute the print process:

**Prior to printing:**

(1) **Level the build plate:** Before the print can commence it is important to ensure the build plate is level with the screen. A tutorial on how to level the SLA printer's base plate can be found here.
(2) **Secure vat/resin reservoir and fill resin:** Once the build plate has been leveled, the vat or resin reservoir must be secured to the printer and filled with resin. Filling the reservoir with resin up to the designated fill line is important to ensure the reservoir does not overflow when the build plate is submerged within it during printing. Once a print is complete, any leftover resin can be used to complete a consecutive print without detaching the reservoir, assuming that there is sufficient resin remaining. The resin bottle should be gently shaken before pouring into the reservoir. If shaken too vigorously, excessive bubbles will form within the resin, which may negatively impact print quality. Before initiating a print, the resin can be allowed to sit within the reservoir for several minutes to allow for any remaining bubbles to dissipate.

Next, the touch screen input can be used to select the model to be printed from the connected flash drive. Once the print initiates, the printer will display the time towards completion. Once the print has completed, the build should be left attached to the build plate for several minutes to allow excess resin to drain from the model into the resin tank.

**Post-print procedure:**

(1) **Removing the Model from Build Plate:** Depending on the exposure time settings, the model's adherence to the build plate can vary. In cases where the model has not adhered strongly to the build plate, the build can be detached using a spatula, which is usually supplied with SLA 3D printers. It is important to note that in some cases a steel spatula is included with the printer. The use of steel implements to remove the model should be avoided as it can easily scratch the build plate. Instead, utilize a plastic spatula to remove the model. If the model is difficult to remove, carefully pour hot water along the build plate, targeting points of contact with the model. The hot water should loosen the model and make it easier to detach.
(2) **Post Processing:** Once the model is removed, clean the build plate and general work area with a paper towel and isopropyl alcohol (IPA). Whilst IPA is the cleaning agent used in this work, other options exist, such as dipropylene glycol monomethyl ether (DPM) and tripropylene glycol monomethyl ether (TPM), which are advantageous if a lower flammability medium is required. Next, carefully remove the support segment off the model. Supports will generally break off by hand, especially when removed within the first few hours after the print has completed. However, in some cases a pair of wire cutters may be required to remove supports. The next step is to wash the model by submerging it in a container filled with IPA. In this work, submersion in IPA was sufficient to adequately clean the residual resin from the internal pore structure of the 3D macromodel presented herein. However, in cases where printed pore structures are highly tortuous or tight, additional processing may be required. In such cases, submersion within a sonic bath containing a cleaning agent and/or flushing the pore system with cleaning agent using a syringe may be employed to remove residually trapped resin. Finally, leave the model in direct sunlight for roughly 30 mins to allow UV rays to cure and harden the build. Alternatively, a dedicated wash and cure station can be used to complete this operation (e.g. Fig. 2). To ensure transparency, the model will typically require sanding and polishing. Sequentially finer grit sandpapers (i.e. 400 to 1500 grit) are used to provide an increasingly refined finish to the macro model. A polishing disk on a rotary multitool or bench top grinder coupled with a polishing compound can be used to achieve the final finish. Surface finishing is typically reserved for the coverslip portion of the macromodel, where achieving a high degree of transparency in critical.

## Method validation

The resin 3D printing method described herein was used to create macromodels based on geologically realistic pore network images, with models serving as the basis for immiscible flooding experiments. These experiments were recorded using a mirrorless digital camera, with images captured at different stages of the flooding procedure. Visual learning projects centered around these images were created to aid undergraduate students in their understanding of various geological concepts through tactile and engaging learning experiences [1]. The study of porous media and petrophysical concepts can be a difficult subject matter to visualize, as the key processes are occluded within the rock volume. However, with the aid of the geological macromodels, fluid transport processes within porous media can be observed directly providing a tangible demonstration of otherwise abstract behavior. For instance, Tong et al. [10] utilized 3D printing to create spherical porous media models and study groundwater flow. Similarly, Anjikar et al. [2] utilized the fused filament 3D printing method to create porous media models with internal pore networks that mimic the reactive properties of sandstone.

Having reduced in cost significantly over the past decade, SLA printing is preferable for macromodels creation as it provides key advantages over other popular 3D printing technologies (e.g. fused depositional modeling: FDM). The most significant advantage within the context of macromodel generation is the high degree of transparency that can be achieved with SLA prints, whereby internal refractions induced by micropores within FDM prints result in translucent models in cases where transparent filaments are used. The porous nature of the solid phase within FDM prints also limits the utility of this technology for experimental fluid imaging, as in contrast to SLA printed media, FDM builds are liable to leak fluids.

A further advantage lies in the ability for SLA printing to achieve a high level of accuracy and resolution in comparison to FDM printing [8,10]. This is a critical feature as macromodels that seek to mimic geological pore networks contain intricate pore structures that require refined print quality for adequate replication. It is worth noting that accuracy can be refined through the print settings at the expense of printing time.

**Fig. 3:**
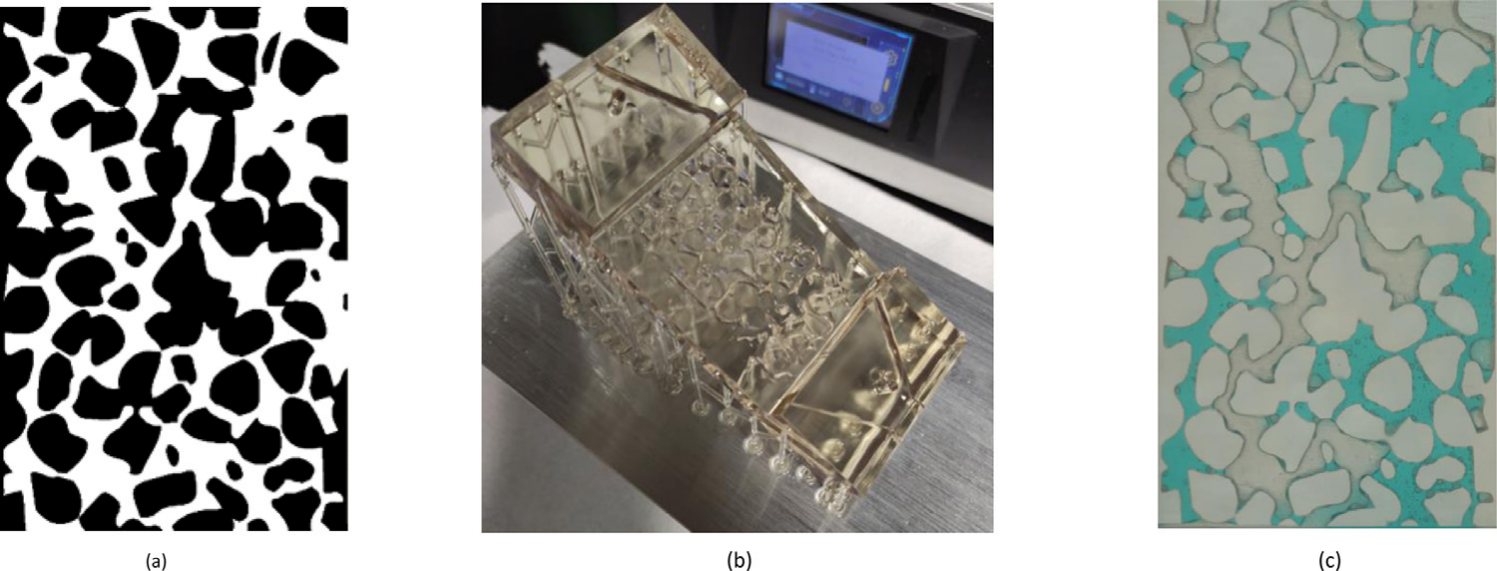
The workflow utilized to create the geological macromodel, we start with (a) binary pore network image, then (b) print the model, and (c) perform the flooding experiment.

**Fig. 4:**
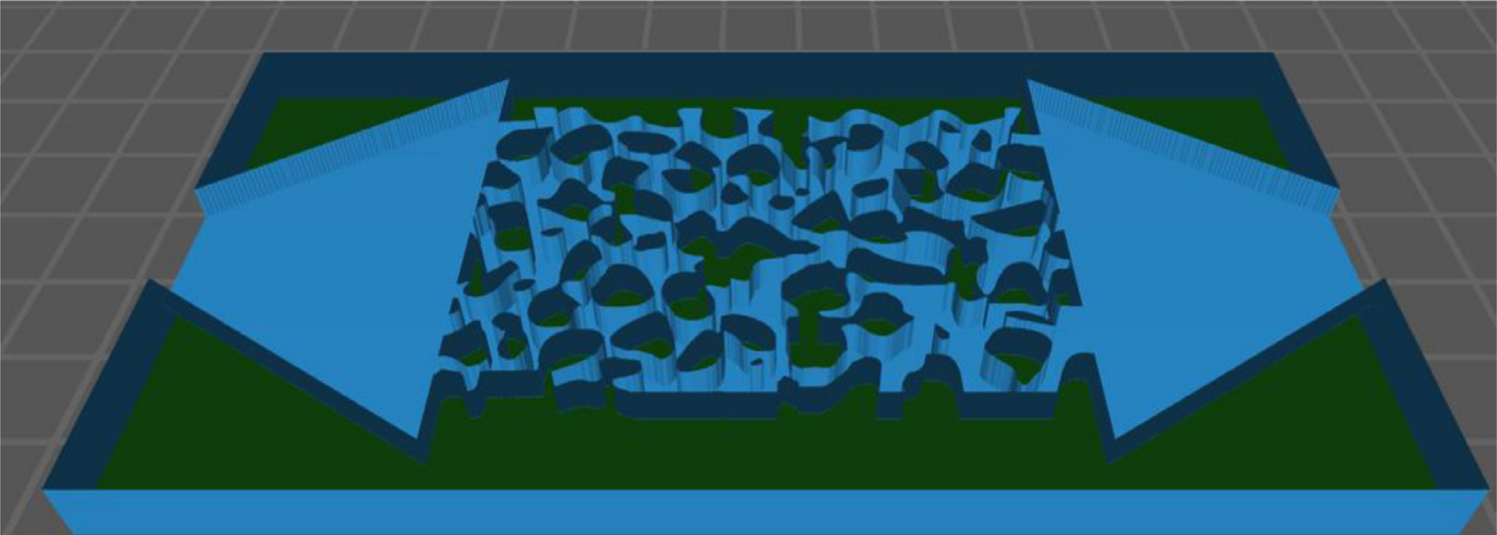
3D view of Macromodel A visualized in the 3D printing Slicer software to highlight details of the 3D pore network.

Fig. 3 summarizes the workflow presented herein to create a geological macromodel utilized in a visual learning project application, with a 3D view of the modelled pore structure displayed in Fig. 4. This view displays the inlet and outlet chambers of the model that were used to inject fluid through the pore network during flooding experiments. The drain holes which can be seen at the ends of both the inlet and outlet chambers allowed for resin to drain out of the pore network and chambers during printing. These drain holes were later sealed with a hot glue gun producing a watertight model. The example macromodel presented herein is one of eight macromodels printed in the project, with each model being based upon a different pore network image [1]. The ability to rapidly replicate the printing procedure for different pore network geometries provides the capacity to study the effect that different pore network properties have upon immiscible fluid transport.

To further enhance the view of the pore network, the surface of the model overlaying the network was sanded in stages with sandpaper of increasing grit size (400, 600, 800 and 1500) and then polished using polishing compound and a polishing disk attached to a rotary tool. Each of the macromodel prints utilized the same print settings which are summarized in Table 1.

**Table 1:** The print settings utilized for each of the 8 resin 3D printed macromodels.

Layer Thickness (mm)	0.05
Normal Exposure Time (s)	2
Bottom Exposure Time (s)	40
Bottom Layers	6
Z Lift Speed (mm/s)	2
Z List Distance (mm)	8

**Fig. 5:**
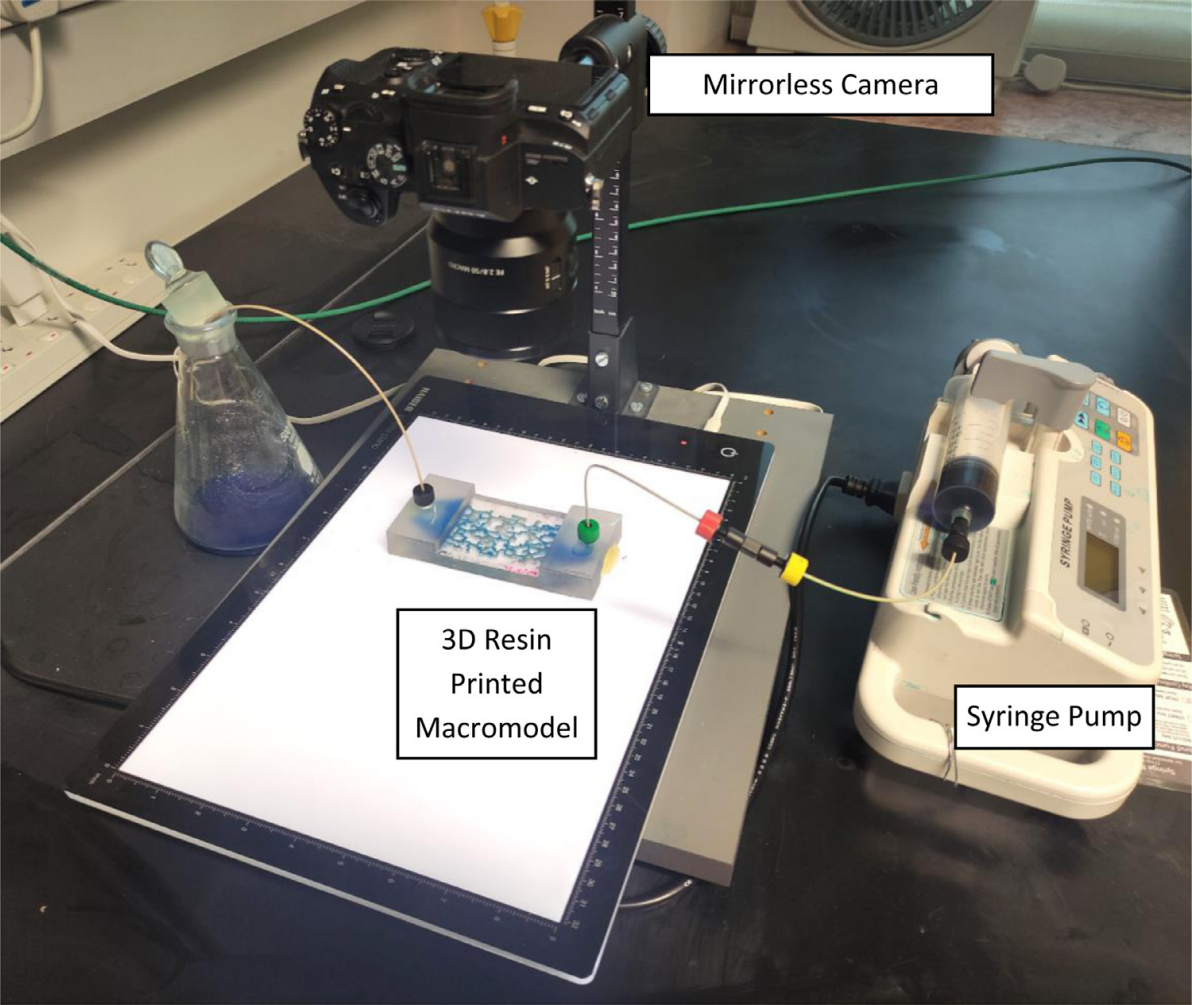
Experimental setup utilized to conduct flooding experiments on the 3D resin printed macromodels.

Once sanded and polished, the inlet of the macromodel was connected to a syringe pump via standard microfluidics 1/16 PEEK flow lines to allow fluid to be pumped through the pore network. The outlet of the model was also connected to a flow line leading to a drainage container to store fluid exiting the macromodel as the experiment is run. It should be noted that the inlets and outlets for this use case are not printed with threaded holes. Inlet/outlet ports are threaded using a tap wrench and 10–32 tap to facilitate connection of the flow lines using one-piece finger tight PEEK fittings. The model is then placed on a light pad directly below a digital camera, mounted in nadir view on a copy stand. The described experimental setup is shown in Fig. 5.

As discussed, the images captured from the flooding experiments were used in a set of learning exercises that allowed students to analyze the images and calculate various geological properties including porosity, fluid saturation, grain size distribution, wettability, and displacement efficiency [1]. These exercises guided students through the analysis of the images using an open-source image processing software (Fiji). The ease at which the 3D resin printing procedure can be reproduced proved to be vital in creating this visual learning project, as it allowed for the effective creation of macromodels with varying pore network properties.

## Declaration of Competing Interest

The authors declare that they have no known competing financial interests or personal relationships that could have appeared to influence the work reported in this paper.

## Appendix Supplementary materials

Image, application 1

Image, application 2

Image, application 3

Image, application 4

Image, application 5

Image, application 6

Image, application 7

**Supplementary material *and/or* Additional information**: (1) Software Slicing Software Download Links: Chitubox, Prusa Slicer. (2) Video on Why 3D Prints Fail. (3) All .stl files of the 8 mode are provided. Image, application 8
